# A Standard Operating Procedure for Protein Extraction From Abdominal Aortic Aneurysm Tissue: Enhancing Proteomics Applications

**DOI:** 10.1002/prca.70030

**Published:** 2025-11-14

**Authors:** Telmo Baltazar, Fábio Trindade, Rita Nogueira‐Ferreira, Rui Vitorino, Rita Ferreira, Pedro Domingues, Adelino Leite‐Moreira, Marina Dias‐Neto, VASCUL‐AID Consortium, Kak Khee Yeung, Vincent Jongkind, Lotte Rijken, Sabrina Zwetsloot, Venkat Ayyalasomayajula, Stefan Smorenburg, Jelmer Wolterink, Henk Marquering, Ivana Išgum, Corrette Ploem, Fabio Catarinella, Regent Lee, Katarzyna D Bera, Juliette Raffort, Fabien Lareyre, Catelijne Muller, Igor Koncar, Ivan Tomic, David Matejevic, Maja Živković, Tamara Djuric, Aleksandra Stankovic, Maarit Venermo, Riikka Tulamo, Mirjami Laivuori, Christian‐Alexander Behrendt, Noeska Smit, Marlies Schijven, Bert‐Jan van den Born, Ronak Delewi

**Affiliations:** ^1^ RISE‐Health, Department of Surgery and Physiology, Faculty of Medicine University of Porto Porto Portugal; ^2^ iBiMED – Institute of Biomedicine, Department of Medical Sciences University of Aveiro Aveiro Portugal; ^3^ LAQV‐REQUIMTE, Department of Chemistry University of Aveiro Aveiro Portugal; ^4^ Department of Cardiothoracic Surgery São João University Hospital Center Porto Portugal; ^5^ Department of Angiology and Vascular Surgery São João University Hospital Center Porto Portugal; ^6^ Department of Surgery, Amsterdam University Medical Center Location University of Amsterdam Amsterdam The Netherlands; ^7^ Department of Surgery, Amsterdam University Medical Center Location Vrije Universiteit Amsterdam Boelelaan Amsterdam The Netherlands; ^8^ Amsterdam Cardiovascular Sciences, Atherosclerosis & Aortic diseases Amsterdam The Netherlands; ^9^ Amsterdam Public Health, Digital Health Amsterdam The Netherlands; ^10^ Department of Applied Mathematics, Technical Medical Centre University of Twente Enschede The Netherlands; ^11^ Department of Biomedical Engineering and Physics, Amsterdam University Medical Center Location University of Amsterdam Amsterdam The Netherlands; ^12^ Department of Radiology and Nuclear Medicine, Amsterdam University Medical Center Location University of Amsterdam Amsterdam The Netherlands; ^13^ Informatics Institute, Faculty of Science, University of Amsterdam Amsterdam The Netherlands; ^14^ Department of Ethics, Law and Humanities, Amsterdam University Medical Center Location University of Amsterdam Amsterdam The Netherlands; ^15^ Chief Medical Information Officer, Brightfish B.V. Hoofddorp The Netherlands; ^16^ Nuffield Department of Surgical Sciences University of Oxford Oxford UK; ^17^ Clinical Chemistry Laboratory, University Hospital of Nice Nice France; ^18^ Institute 3IA Côte d'Azur, Université Côte d'Azur Nice France; ^19^ Université Côte d'Azur, CNRS, UMR7370, LP2M Nice France; ^20^ Department of Vascular Surgery Hospital of Antibes Juan‐les‐Pins Antibes France; ^21^ ALLAI Amsterdam The Netherlands; ^22^ Medical Faculty University of Belgrade Belgrade Serbia; ^23^ Clinic for Vascular and Endovascular Surgery, Clinical Center of Serbia Belgrade Serbia; ^24^ Laboratory for Radiobiology and Molecular Genetics, VINCA Institute of Nuclear Sciences ‐ National Institute of the Republic of Serbia, University of Belgrade Belgrade Serbia; ^25^ Department of Vascular Surgery Helsinki University Hospital Helsinki Finland; ^26^ University of Helsinki Helsinki Finland; ^27^ Department of Vascular and Endovascular Surgery Asklepios Clinic Wandsbek, Asklepios Medical School Hamburg Germany; ^28^ Department of Informatics University of Bergen Bergen Norway; ^29^ MMIV, Department of Radiology Haukeland University Hospital Bergen Norway; ^30^ Amsterdam Gastroenterology and Metabolism Amsterdam The Netherlands; ^31^ Department of Public and Occupational Health & Department of Vascular Medicine Amsterdam University Medical Center, Location University of Amsterdam Amsterdam The Netherlands; ^32^ Department of Cardiology, Amsterdam University Medical Center Location University of Amsterdam Amsterdam The Netherlands; ^33^ Amsterdam Cardiovascular Sciences Amsterdam The Netherlands

**Keywords:** abdominal aortic aneurysm, aortic tissue homogenization, protein extraction, proteomics, SOP

## Abstract

**Purpose:**

The identification of putative biomarkers for AAA can be achieved through shotgun proteomics. However, tissue heterogeneity hampers its reproducible homogenization and protein extraction. Thus, we aimed to optimize a protocol to maximize protein yield and develop an SOP to foster reproducibility and accelerate translation of proteomics findings.

**Experimental Design:**

Using a bead‐beating homogenization method, we compared the effect of beads’ size, extraction cycles, beads‐to‐tissue mass ratio, lysis buffer volume, and chemistry on protein yield and/or qualitative and quantitative parameters of proteomics analysis (identifications, sequence coverage, coefficient of variation, functional enrichment analysis).

**Results:**

Optimal conditions for protein extraction were achieved using 1.4 mm beads in two homogenization cycles, with a bead‐to‐tissue mass ratio of 30:1 and 20 µL of lysis buffer per mg of tissue. As for the buffer chemistry, RIPA is recommended to attain greater sequence coverage, while HEPES and Urea/thiourea are preferred when quantification performance is a priority. The SOP was applied to characterize the AAA tissue proteome, and key AAA pathogenesis‐related pathways were highlighted by bioinformatic analysis.

**Conclusions and Clinical Relevance:**

The SOP is well‐suited for identifying and quantifying aneurysmatic tissue proteins and can be applied to accelerate the translation of putative biomarkers into clinical diagnostic/prognostic tools.

**Summary:**

- Abdominal aortic aneurysm (AAA) is a life‐threatening, non‐communicable disease that remains underdiagnosed and poorly understood within the medical community. Furthermore, there is a lack of an effective medical therapy, aside from surgical intervention, that compels clinicians to address general cardiovascular risk factors for disease management.
- Thus, fishing putative new biomarkers and therapeutic targets from aneurysmatic tissue using untargeted proteomic approaches has emerged as a relevant strategy for the development of tools for earlier diagnosis, effective disease management, and a deeper understanding of the pathophysiology of AAA.
- However, given the heterogeneity of AAA tissue, the reproducibility of the results may be partially affected by the absence of a standardized method for protein extraction while ensuring high efficiency in protein yield. Therefore, this study aimed to address a key bottleneck in proteomic analysis of aneurysmatic study—heterogeneity in sample processing.
- Herein, we report the optimization of a protocol for AAA tissue homogenization and maximize protein extraction. A standard operating procedure (SOP) to process AAA tissue toward downstream proteomics applications is shared to enable more reliable and comparable data across studies and ultimately bolster the translation of tissue proteomics into clinically relevant tools for vascular medicine.

**Abbreviations**
AAA: abdominal aortic aneurysm
BP: biological process
CC: cellular component
CV: coefficient of variance
ECM: extracellular matrix
EC: extraction cycle
FA: formic acid
LB: lysis buffer
LFQ: label‐free quantification
MF: molecular function
MMP‐12: matrix metallopeptidase 12
MW: molecular weight
OAR: open aortic repair
R‐HSA: reactome‐*Homo sapiens*
RIPA: radio‐immunoprecipitation assay
SC: sequence coverage
SOP: standard operating procedure

1

Abdominal aortic aneurysm (AAA) is a complex and degenerative disease, characterized by the progressive destruction of aortic wall integrity due to the focal expansion of the abdominal aorta, typically diagnosed on imaging when the aortic diameter exceeds 30 mm [1]. This condition is usually asymptomatic, but once the aneurysm ruptures, it could be life‐threatening and even fatal without successful surgical repair, being associated with a mortality rate of 65%–85% [2]. Despite the advances in surgical interventions, currently, no clinically validated treatments for the management of aortic degeneration and the control of rupture risk are available, besides endovascular aortic repair and open aortic repair (OAR) [2, 3]. Therefore, a key remaining priority in this context is the development of novel successful therapies, mainly targeting early‐stage aneurysms to limit the growth and expansion rates and, consequently, prevent their rupture. In addition, the pathophysiology of AAA formation, enlargement, and rupture remains incompletely understood, and target disease biomarkers for AAA diagnosis and interventional decision‐making are lacking [4, 5]. A potential starting point for the discovery of non‐invasive therapies to treat, or even prevent, AAA progression is to invest in the comprehension of molecular mechanisms underlying AAA pathophysiology.

Over the past decades, mass spectrometry (MS)‐based proteomics technology has become revolutionary and widely accessible, allowing the analysis of complex protein mixtures and the identification and quantification of target molecules that have deepened our understanding of pathological mechanisms [6]. Particularly, proteomics of human plaques’ extracts has been applied to the discovery of potential mechanisms underlying atherosclerosis disease, showing promise for powerful applications in AAA research and for unveiling potential new biomarkers [7]. However, despite the increasing number of vascular proteome studies reported in the literature, an SOP for AAA tissue proteomics is lacking. Compared to other tissues, such as hepatic or cardiac muscle tissues, AAA tissue presents a high degree of heterogeneity that arises from the presence of degraded elastin in the extracellular matrix (ECM), fibrotic areas, inflammatory cell infiltrates, and, in advanced stages of AAA, atherosclerotic regions enriched with lipid and/or calcified deposits [8]. Such compositional variability results in inconsistent mechanical properties, making the tissue highly resistant to physical disruption using standard homogenization methods. In previous studies, a variety of methodological strategies have been employed to address the challenges of protein extraction from AAA tissue [9, 10, 11]. For instance, Andersen et al. compared the proteomes of the aortic wall and intraluminal thrombus using detergent‐based extraction combined with ultrasonication for tissue processing. Didangelos et al. employed a solubility‐based subfractionation approach following subsequential treatment with NaCl, SDS, and guanidine‐HCl to target the ECM subproteome and provide a deeper insight into structural remodeling. To characterize the proteomic profile of calcified AAA tissue, Matsumoto et al. applied an urea‐based lysis buffer with sonication for protein solubilization before iTRAQ‐based MS analysis. These studies exemplify the diverse protein extraction methods used to characterize AAA proteome, but efforts to optimize the protocols and improve reproducibility are minimal. Therefore, we propose a methodology employing a bead‐beating system with zirconium dioxide (ZrO_2_) beads to extract protein from AAA wall. Zirconia beads are highly effective for the disruption of hard, dense, and fibrotic structures present in vascular tissues, as we have demonstrated for calcified aortic tissue [12]. Compared with conventional approaches, including grinding‐based or ultrasonication methods, this system enhances experimental reproducibility by reducing operator‐dependent variability and provides scalability, particularly advantageous for high‐throughput proteomic analyses. For these two main reasons, we aimed to optimize a bead‐beating method and develop an SOP to foster reproducibility in the AAA proteome analysis.

The following methodology was employed to maximize tissue homogenization and protein extraction.

Human AAA tissue samples, collected during OAR, were sourced from our local biobank. In total, five AAA biological samples (labeled with A to E) were used through the experiments, four in the optimization phase (A to D) and three in the proof‐of‐concept experiment (B, D, and E). This study followed the principles stated in the Declaration of Helsinki, and the ethics committee of the Centro Hospitalar Universitário de São João approved the protocol (reference CE‐159‐2014). Informed consent was obtained from all patients. After surgical resection, any existing thrombi were carefully dissected from the aortic wall, to avoid bias. The aortic samples were kept at 4°C and transported to the laboratory within 2 h of collection. The samples were washed with PBS to remove any remaining blood contaminants and minor clots, flash‐frozen in liquid nitrogen, and stored at −80°C until further processing.

In order to achieve the optimal conditions for protein extraction, several experiments were performed aiming to obtain the highest protein yield and a sharp protein electrophoretic profile. For the experimental design of the homogenization protocol, we based on an SOP previously developed by our research group for calcified aortic valves [12], given a certain degree of histological similarity, including atherosclerotic‐like features. Hence, we established the use of a bead‐beating system (Precellys Evolution Touch 23405‐300‐RD001, Bertin Technologies, Montigny‐le‐Bretonneux, France) employing zirconium dioxide beads (Bertin Technologies, Montigny‐le‐Bretonneux, France) for tissue fragmentation, in combination with a lysis buffer (LB) to maximize protein solubility and integrity for downstream analyses. To maximize the protein yield we optimized the following variables: a) beads size (1.4 vs. 2.8 mm of diameter); (b) the number of extraction cycles (one, two, or three), with an extraction cycle (EC) defined as a single event of homogenization prior to centrifugation; (c) the beads mass‐to‐tissue mass ratio (20, 30, or 40×); (d) LB volume‐to‐tissue mass ratio (10, 15, or 20 µL/mg tissue); and lastly, (e) LB's chemistry, utilizing three distinct formulations (Radio‐Immunoprecipitation Assay (RIPA), Urea/thiourea, and HEPES). The detailed composition of each buffer is presented in Table 1. A step‐by‐step optimization process was followed, where variables defined as optimal were standardized in the subsequent experiments. The final optimized protocol is provided as an SOP in Supporting File S1.

**TABLE 1:** Composition of the lysis buffers.

Buffer	Buffer composition	Reference
RIPA	25 mM Tris‐HCl, 150 mM NaCl, 1% NP‐40, 1% sodium deoxycholate, 0.1% SDS, pH 7.6	Trindade et al. [12]
Urea/thiourea	8M urea, 2M thiourea, 45 mM Tris‐HCl, 4% CHAPS, pH 8.0	Adapted from Sung et al. [13], Herrington et al. [14]
HEPES	25 mM HEPES, 1% sarcosyl, pH 7.4	Adapted from Malaud et al. [15]

*Note:* In any case, % refers to m/V.

The selection of the LBs was based on insights from previous studies [12, 13, 14, 15] and their established application in tissue proteomics, particularly for atherosclerotic tissue. Their suitability for MS analysis was also a key consideration in the selection process. All buffers were prepared or completed in‐house and were supplemented with 1 mM EDTA (EDTA disodium salt dihydrate, ≥99% Merck‐Millipore, Darmstadt, Germany), a protease inhibitor cocktail (Halt PIC, EDTA‐free 100×, Thermo Fisher Scientific), and a phosphatase inhibitor cocktail (PhosSTOP, Roche, Mannheim, Germany), at the recommended concentration by the manufacturer, to ensure protein stability and prevent degradation during the extraction process.

After homogenization, in all cases, the protein lysates were centrifuged at 13680 *× g*, for 15 min at 4°C (Mikro 200 R centrifuge, Hettich Zentrifugen, Tuttlingen, Germany), and the protein‐rich supernatants were stored at −80°C until further processing. Protein concentration was estimated using a detergent‐compatible kit (DC kit, Bio‐Rad Laboratories, Hercules, CA, USA). Samples were diluted 10 × and directly quantified, except in the last test, comparing different LBs. In this case, an additional step of protein precipitation with acetone (9:1 acetone:lysate V/V) overnight at −20°C was required, given the incompatibility of highly concentrated urea buffer (>4 M) with the DC method. Essentially, after overnight precipitation, samples were centrifuged at 14000 *× g* for 30 min, at 4°C, the supernatants discarded, and any residual urea and thiourea was removed by carefully washing the pellets with 90% ice‐cold ethanol in the same proportion (9:1). This precipitation step was also applied to RIPA and HEPES lysates to ensure consistency in sample preparation and to eliminate any potential bias in protein quantification. The protein pellets were resolubilized in 0.1% SDS. BSA was used as the standard for protein quantification.

The protein extracts were separated in SDS‐PAGE gels following the Laemmli procedure [16]. Essentially, 40 µg of protein from each sample was diluted to the same concentration and mixed with Laemmli buffer, incubated for 5 min at 95°C, and loaded into discontinuous Tris‐HCl gels (4% and 12% stacking and resolving gels, respectively). The proteins were separated under reducing and denaturing conditions using the Bio‐Rad SDS‐PAGE system, at 120 V for 10 min, and then at 200 V for 40 min. The gels were then incubated in a fixation solution (40% methanol, 10% acetic acid) for 30 min, stained with 0.12% Colloidal Coomassie Blue G250 in 20% methanol overnight, and distained with deionized water until optimal contrast was achieved. Gels were scanned with the ChemiDoc system and analyzed with ImageLab (version 6.0.1, BioRad Laboratories, Hercules, CA, USA) for automatic detection of protein bands.

For proteomic characterization and analysis, each AAA protein extract was mixed with loading buffer (0.5 M Tris‐HCl pH 6.8, 4% (m/V) SDS, 15% (V/V) glycerol, 100 mM DTT, 0.04% (m/V) bromophenol blue) and the proteins were denatured and reduced by boiling for 5 min at 95°C, and alkylated with a 40% acrylamide solution (1:15 V/V). Then, the samples were loaded in a 12% SDS‐PAGE gel, prepared according to Laemmli [16]. A Short‐GeLC approach was followed, where the proteins were separated by SDS‐PAGE for 10 min at 120 V and 10 min at 200 V, and stained with Coomassie Brilliant Blue G‐250. For proteomic analysis, 40 µg of protein was loaded into the gel, and each lane was carefully excised from the gel and divided into five fractions. *In‐gel* digestion was performed according to Shevchenko et al. [17] with a few modifications. Gel fractions were washed and distained with 25 mM ammonium bicarbonate (NH_4_HCO_3_), followed by 25 mM NH_4_HCO_3_/ACN (1:1), and ACN (VWR Chemicals). Proteins’ cysteine residues were reduced with 10 mM DTT and alkylated with 55 mM iodoacetamide in the dark, followed by a second washing step of the gel fractions under the same conditions. Gel pieces were dried in the incubator at 56°C, and rehydrated in a digestion buffer containing sequencing‐grade modified trypsin in 50 mM NH_4_HCO_3_. Enzymatic digestion was performed with trypsin (90057; Pierce Trypsin Protease MS‐Grade) at an enzyme:substrate ratio of 1:30 (m/m), overnight (17 h) at 37°C. Tryptic peptide extraction was done by washing the extracts with 5% formic acid (FA, Fluka), and 5% FA in ACN (1:1). The tryptic peptides were lyophilized in a vacuum concentrator (SpeedVac, Thermo Savant), resuspended in 1% FA solution (V/V), centrifuged at 15,000 *× g* for 10 min, and 2 µL of the supernatant were collected and injected into a C18 PepMap column, for LC‐MS/MS analysis.

Samples were analyzed on a NanoLC System with a Q‐Exactive Hybrid Quadrupole‐Orbitrap (Thermo Fisher Scientific, Bremen) through the EASY‐spray nano ESI source (Thermo Fisher Scientific, Bremen) coupled to an Ultimate 3000 (Dionex, Sunnyvale, CA) HPLC system. The trap (5 mm × 300 µm of internal diameter (i.d.)) and the EASY‐spray analytical (150 mm × 75 µm) columns used were C18 PepMap (Dionex, LC Packings) with a particle size of 3 µm. Peptides were trapped at 30 µL/min in 96% solvent A (0.1% FA), and the elution was achieved with solvent B (0.1% FA/80% ACN (V/V)) at 300 nL/min. The 92‐min gradient used was performed as follows: 0–3 min, 96% solvent A; 3–70 min, 4%–25% solvent B; 70–90 min, 25%–40% solvent B; 90–100 min, 90% solvent B; 101–120 min, 96% solvent A. The mass spectrometer was operated at 1.7 kV in the data‐dependent acquisition mode. A MS2 method was used with a FT survey scan from 400 to 1600 m/z (resolution 70,000; automatic gain control, AGC, target 1E6), and the 10 most intense peaks were submitted to higher‐energy collisional dissociation fragmentation (resolution 17,500; AGC target 5E4, normalized collision energy 28%, max. injection time 100 ms, dynamic exclusion 35 s).

MaxQuant (version 2.0.0.0, Max Planck Institute of Biochemistry, Planegg, DE) was used for protein identification and for label‐free quantification (LFQ). MS/MS spectra were matched with the UniProt (TrEMBL and Swiss‐Prot) *Homo sapiens* database (version of June 2024) using the Andromeda search engine. Regarding the database search parameters, methionine oxidation, N‐terminal protein acetylation, and phosphorylation were considered variable modifications, while cysteine carbamidomethylation was set as a fixed modification. Fragment ion mass and precursor mass tolerances were set at 0.15 Da and 20 ppm, respectively. Up to two missed cleavages were allowed, and the minimum peptide length was set to seven amino acids. FDR for protein and peptide identification was kept to 1% and MaxQuant‐identified contaminants were excluded.

In order to infer the processes and pathways that can be monitored with our method, a functional enrichment analysis of GO terms on biological processes (BPs), molecular functions (MFs), and cellular components (CCs) as well as of Reactome pathways was performed with the ShinyGO tool (version 0.81, assessed in January 2025, South Dakota State University, USA) [18]. In both cases, a minimum number of two genes was allowed, and the FDR was set to 0.05.

All the data are expressed as mean ± SD. The statistical significance of the differences was assessed using distinct statistical tests: (a) paired Student's *t*‐test, to evaluate differences in protein concentration obtained in the first experiment; and (b) one‐way or two‐way ANOVA, followed by Tukey's multiple comparisons post hoc test, selected based on the number of independent variables analyzed for the subsequent experiments. These statistical tests were performed in GraphPad Prism (8.0.2 version, GraphPad Software, San Diego, California, USA), and the level of significance was set at 5% (*p* value < 0.05).

The accumulation of atherosclerotic plaque, ECM, and the extent of calcification, contributing to different degrees of tissue stiffness and vulnerability to rupture, and other regional structural differences, such as in arterial wall thickness, contribute to the heterogeneity of AAA tissue [19]. This factor poses challenges for a reproducible molecular profiling of these disease‐informative biopsies, including through proteomic characterization, slowing the translation of the findings to clinically relevant biomarkers and therapeutic targets. Given the impossibility to bypass the biological variability of aneurysmatic aortas, an effort should be made to optimize and standardize the homogenization protocol and eliminate or significantly reduce technical variance. In this sense, we aimed to develop an SOP to be used across different laboratories and foster the reproducible analysis of the AAA tissue proteome, ultimately contributing to a better understanding of the disease's pathophysiology and accelerating the discovery of novel biomarkers and therapeutic targets.

In order to test the effect of beads’ size and the number of homogenization cycles on protein yield, six fragments (m ≈ 15 mg) from the same biological sample (A) were processed with 1.4 mm (medium‐sized) versus 2.8 mm (large) ZrO_2_ beads for one, two, and three ECs. Although not statistically significant, the protein extraction tended to be more efficient with smaller beads (5.86 ± 2.63 µg/µL) than with larger beads (5.08 ± 1.53 µg/µL, *p* value = 0.5433), regardless of the number of ECs (Figure 1). In the bead‐beating system, while the high rate of collisions between beads and the tissue, in a closed system, increases the efficiency of the protein extraction as compared to the traditional Potter–Elvehjem Pestle system [12], the accumulation of kinetic energy generates heat that may degrade proteins that are more labile. For that reason, we used short cycles (30 s) and rested the samples on ice for 5 min between cycles. Since we did not observe any significant increase in protein concentration with an increasing number of cycles, we decided to use up to two ECs in the following experiments.

**FIGURE 1:**
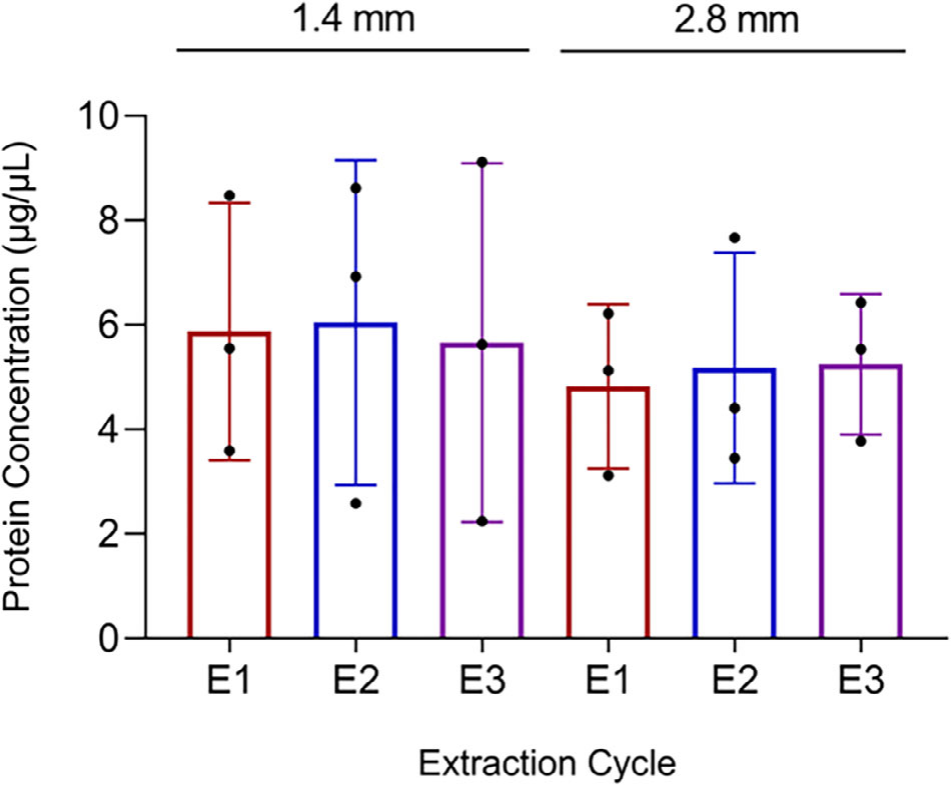
Protein concentration (µg/µL) of the tissue lysates obtained using medium (1.4 mm) and large‐sized (2.8 mm) zirconium dioxide beads, in up to three ECs. Plot bars represent the mean ± standard deviation. Significance was established for a *p* value < 0.05.

Based on the previous results, and to ensure reproducibility, we repeated the experiment to compare the efficiency of protein extraction using one versus two ECs, while comparing the use of 1.4 and 2.8 mm beads. Given the fact that, often times, human biopsies are minute or have to be divided for different experiments, from this experiment onward, we privileged the comparison of the methods in regard to the protein yield (mg protein/mg tissue, %). The results from the second EC conclusively demonstrated an improvement in protein yield (2.24 ± 0.15% after the 2^nd^ EC versus 0.45 ± 0.19% after the 1^st^ EC, *p* value < 0.0001, for 1.4 mm; 1.62 ± 0.36% after the 2^nd^ EC versus 0.29 ± 0.17% after 1^st^ EC, *p* value = 0.0012, for 2.8 mm), regardless of beads’ size (Figure 2A). When comparing the extraction using beads of different sizes, an increase in protein yield with smaller beads was apparent, and this can be explained, at least in part, by the observation of small tissue remnants when using 2.8 mm beads, evidencing an incomplete tissue homogenization, even after the 2^nd^ EC (for two EC's: 1.4 mm, 2.24 ± 0.15%; 2.8 mm, 1.62 ± 0.36%; *p* value = 0.0482). Moreover, an SDS‐PAGE profile analysis of the lysates (Figure 2B) evidenced sharp band patterns, consistent across all samples. Thus, we concluded that under the established conditions, protein extraction efficiency is maximized using 1.4 mm beads and two ECs.

**FIGURE 2:**
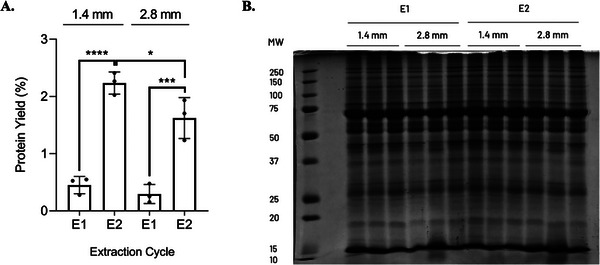
(A) Protein yield [(mg protein/mg tissue) × 100%] from the tissue lysates obtained using 1.4 mm versus 2.8 mm zirconium dioxide beads, after one and two ECs. Plot bars represent the mean ± standard deviation. Significance was established for a *p* value < 0.05 (*p < 0.05; ***p < 0.005; ****p < 0.0001). (B) SDS‐PAGE profile of the tissue lysates. Fifty micrograms (µg) of protein were loaded in the gel. One biological sample (A) was used in this test. MW: Molecular weight; E1: 1^st^ EC; E2: 2^nd^ EC.

After establishing the optimal conditions for bead size and the number of ECs, we sought to assess the impact of increasing the mass of beads and the volume of LB on protein extraction performance. For these experiments, two distinct biological tissue samples were utilized: one sample for testing the first variable, and both samples for the second variable; in any case, ≈15 mg of tissue was used. No modifications were made to the previously optimized protocol, except for the conditions under investigation, which included: (a) increasing the bead amount to 20, 30, or 40 times the tissue mass, and (b) varying the volume of lysis buffer relative to tissue mass (10, 15, or 20 µL/mg). The protein yield obtained under these conditions is shown in Figure 3.

**FIGURE 3:**
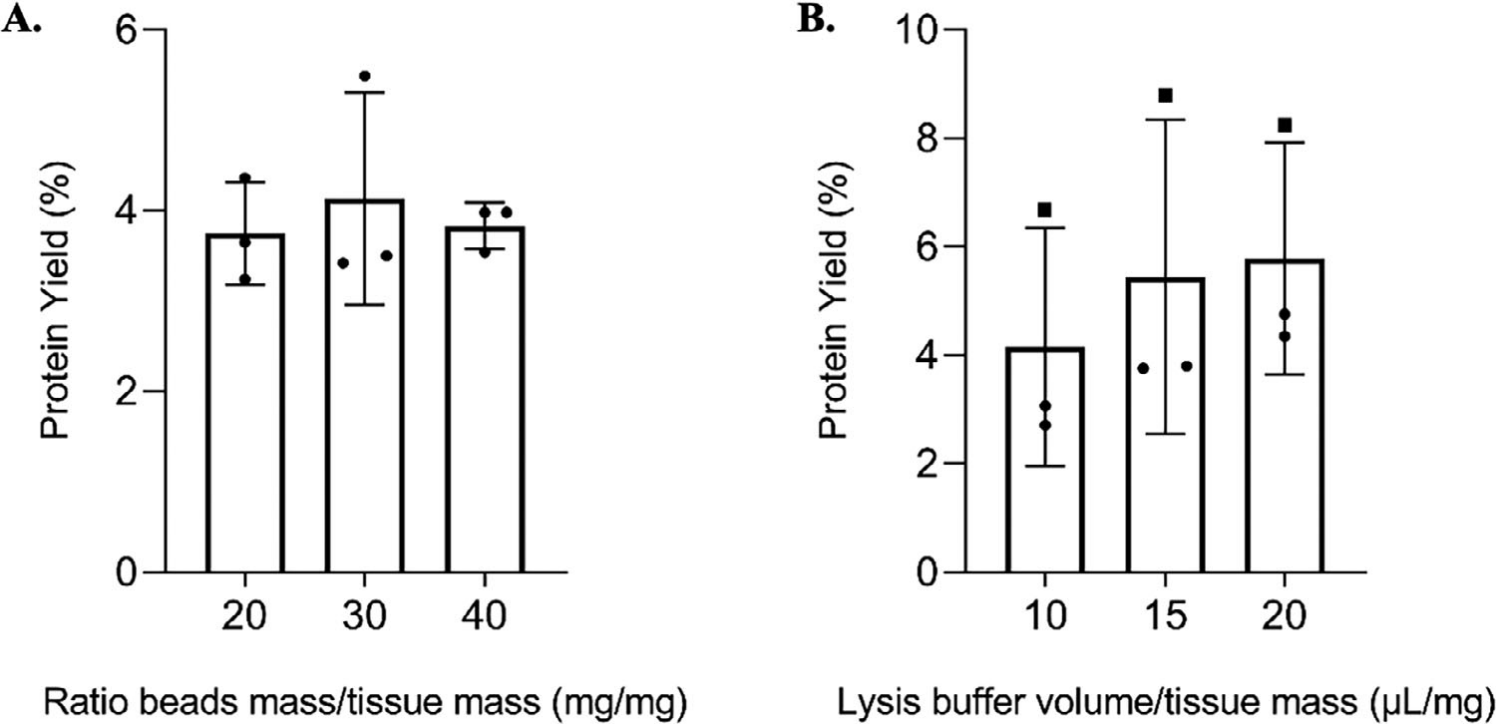
Comparison of the protein yield [(mg protein/mg tissue) × 100%] when increasing the bead mass‐to‐tissue mass ratio (mg/mg) (A) and the lysis buffer volume‐to‐tissue mass ratio (µL/mg) (B) Different dot shapes correspond to distinct biological samples (A—•; B—■) Plot bars represent the mean ± standard deviation, and statistical significance was set at *p* value < 0.05.

Regarding the effect of bead quantity on protein extraction efficiency, although the observed differences did not reach statistical significance, an improvement in protein yield was noted when using a bead quantity 30 times the tissue mass (4.14 ± 1.18%), compared to the other proportions tested (20 × , 3.75 ± 0.57%; 40 × , 3.83 ± 0.26%) (Figure 3A). Based on these results, we established the optimal bead‐to‐tissue mass ratio at 30. Similarly, it was notorious a trend for an increase in protein yield with an increase in LB volume. Specifically, doubling the volume of lysis buffer resulted in an average protein yield that was 1.4 times greater (10 µL/mg, 4.16 ± 2.20%; 20 µL/mg, 5.78 ± 2.14%; Figure 3B). This probably did not reach significance given the use of two different biological samples, which is the main factor for extraction variability. In any case, we can see the upward trend of increased yield with higher volumes of LB, irrespective of the sample (depicted with a circle and a square). These findings suggest that increasing the LB volume beyond 20 µL/mg tissue could further enhance protein extraction efficiency. However, the protein concentration decreases with the increase in lysis buffer volume, resulting in more diluted extracts, which pose challenges for downstream analyses. In extreme cases, this dilution may demand protein precipitation, which can lead to losses during the process and introduce greater variability and heterogeneity into the samples. The SDS‐PAGE profiles for the variables tested are presented in Figures S1 and S2 (Supporting File S2). Remarkably, the electrophoretic patterns were very reproducible among technical replicates, and the differences between lanes were only evident between biological duplicates (A and B).

The composition of the LB is critical for the efficiency of protein extraction from biological tissues, particularly in complex and heterogeneous matrices like atherosclerotic arterial tissue, determining the proteins that remain soluble and those that precipitate out. Besides, the selection of an appropriate LB chemistry is crucial to ensure the compatibility of the LB with tissue sample preparation and downstream applications. Apart from the RIPA buffer, other LBs are commonly used in tissue proteomics (Table 1), particularly the Urea/thiourea or HEPES buffers. To evaluate the impact of LB chemistry on protein extraction performance, these three buffers with distinct formulations were tested under the previously optimized conditions. In this experiment, three biological replicates were used for each LB tested, and the protein quantification was performed using the DC assay, with an additional protein pre‐precipitation step, to ensure compatibility with the method. The results of the protein yield and the SDS‐PAGE profile obtained are presented in Figure 4.

**FIGURE 4:**
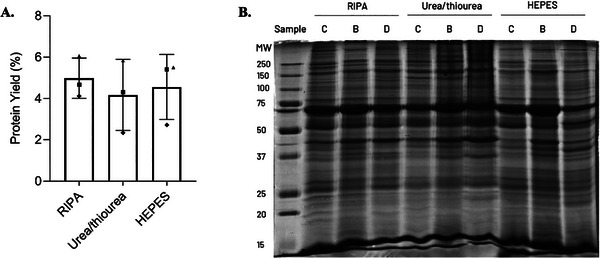
Effect of LB composition on protein extraction. (A) Protein yield [(mg protein/mg tissue) × 100%] obtained with the use of RIPA, Urea/thiourea, and HEPES buffers. (B—■; C—▲; D—◆) Plot bars represent the mean ± standard deviation, and statistical significance was set at *p* value < 0.05. (B) SDS‐PAGE profile of the AAA tissue lysates obtained after the extraction with the RIPA, Urea/thiourea, and HEPES buffers. Twenty micrograms (µg) of protein were loaded in the gel. B, C, and D refer to different biological AAA samples.

The use of RIPA, Urea/thiourea, and HEPES buffers achieved an average protein yield of 4.98 ± 0.97%, 4.17 ± 1.72%, and 4.56 ± 1.58%, respectively. No significant statistical differences were found (*p* value = 0.463). The extraction with RIPA guaranteed about 1.2 and 1.1 times as much protein as Urea/thiourea and HEPES (Figure 4A), which is not relevant on a technical standpoint; nevertheless, extractions were more consistent (lower standard deviation). Thus, the use of RIPA buffer is recommended for protein extraction from AAA tissue when reproducibility is key. Curiously, when comparing the electrophoretic protein profile of the same biological sample extracted with different LBs, few changes in the lane profile seem to be visible. Remarkably, a high number of well‐defined bands across all samples and LBs was found, showing that the homogenization protocol is fit for different LBs (Figure 4B). Thus, using Urea/thiourea or HEPES buffers is also adequate, and the choice of LB should be determined according to the downstream techniques. For instance, for phosphoproteomics, the use of Urea/thiourea LB is usually recommended [20].

Based on the optimal conditions, we have designed an SOP, available for consultation in Supporting File S1, with a detailed description of all reagents, materials, and steps involved in tissue homogenization and protein extraction. Adhering to this SOP will increase the reproducibility of the protein analysis, accelerating clinical translation of the findings. An overview of the optimized experimental conditions for tissue homogenization and protein extraction from AAA tissue is depicted in Figure 5.

**FIGURE 5:**
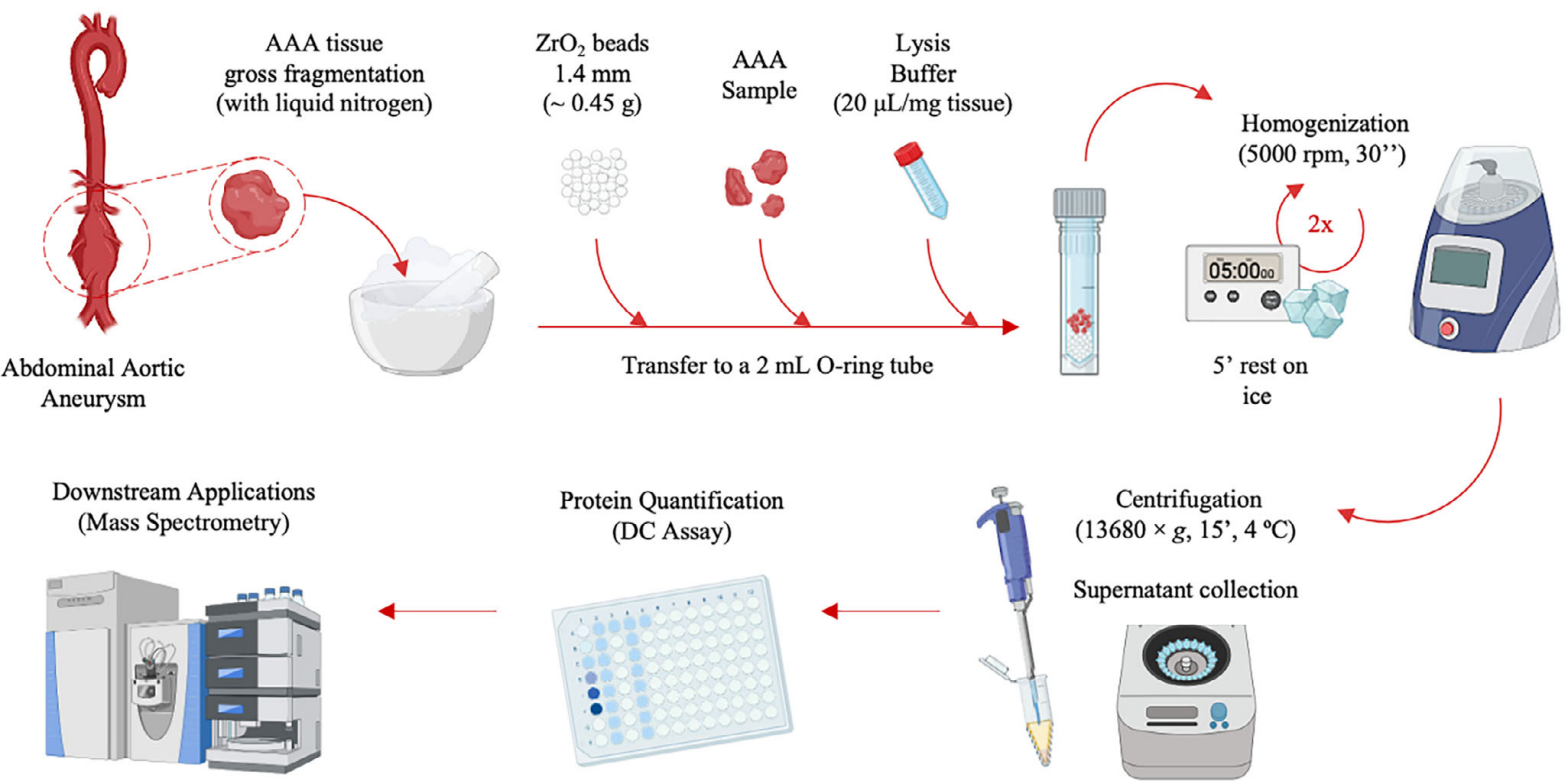
Overview of the workflow for the AAA tissue homogenization and protein extraction (Created with BioRender.com).

Following the optimization of the protocol for tissue homogenization and protein extraction, its suitability for downstream proteomics applications was systematically evaluated in a pilot study. Three samples from three distinct AAA patients (B, D, and E) were homogenized with different LBs (RIPA, Urea/thiourea, and HEPES). The standardized SOP workflow (Figure 5) was applied for the homogenization and protein extraction of the nine samples. The protein lysates were subjected to SDS‐PAGE for preliminary separation and visualization of protein bands, followed by tryptic digestion *in‐gel*, and the obtained peptides were analyzed by high‐resolution MS, enabling the identification and relative quantification of the proteins based on their peptide spectral intensities and abundance ratios. Then, the use of different LBs was compared in regard to important quality control parameters in proteomics, such as the number of proteins identified versus the number of assigned peptides, sequence coverage, coefficient of variation, as well as from the translational perspective, that is, relevance of the mapped GO terms and pathways in relation to AAA pathophysiology.

By LC‐MS/MS analysis, a total of 1645 proteins were identified across all LBs, after filtering the data for reverse database hits and potential contaminants. We then evaluated the number of proteins identified with 0, 1, 2, or ≥ 3 peptides (Figure 6A–C). In total, 717, 1100, and 1144 proteins with at least two peptides were identified, using the RIPA, Urea/thiourea, and the HEPES buffers, respectively. The proportion of confidently identified proteins (i.e., with ≥2 peptides) was higher for HEPES (70%) and Urea/thiourea (67%) than for RIPA buffer (43%) (Figure 6). When analyzing the Venn diagram (Figure 6D) presenting the distribution of the proteins obtained with each extraction, a large portion (roughly half) of protein identifications was in the intersection of the three buffers, with 646 commonly identified proteins. However, it is visible a higher number of exclusively identified proteins (with at least two peptides) with HEPES extraction (*n* = 160, corresponding to 14% of the total set of protein identifications with this LB), followed by Urea/thiourea (*n* = 132, 12%), and RIPA (*n* = 15, 2%) buffers. This suggests that HEPES, closely followed by Urea/thiourea buffer, improves the discovery potential of MS.

**FIGURE 6:**
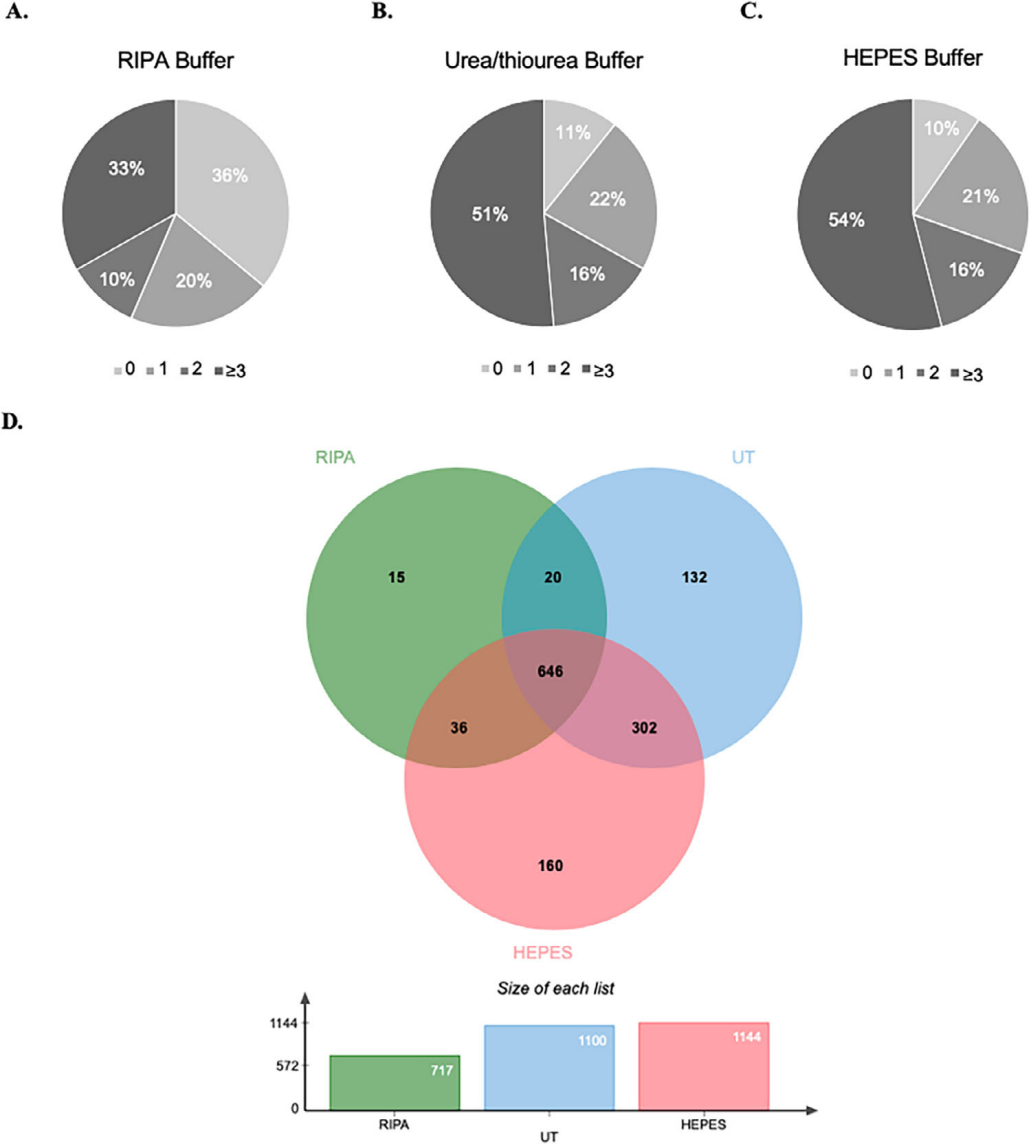
Protein identification performance by LC‐MS/MS. Pie charts illustrating the distribution of proteins identified with 0, 1, 2, or ≥ 3 peptides combining the three biological replicates, using RIPA (A), Urea/thiourea (UT) (B), and HEPES (C) buffers. Proteins identified with 0 peptides reflect that the protein has been detected or annotated in the dataset, but no unique peptide sequences corresponding to that protein were identified in the sample. (D) Venn diagram depicting the overlap in protein identifications for the RIPA, Urea/thiourea, and HEPES LBs. Only proteins identified with at least two peptides were selected. In total, 646 proteins were commonly identified between the three extraction conditions, and 15, 132, and 160 proteins were exclusively identified for extraction with RIPA, Urea/thiourea, and HEPES, respectively.

Aside from the protein identification performance, we aimed to evaluate the quantification performance of each buffer. For this analysis, only the proteins quantified with at least two unique peptides were considered. Only proteins effectively quantified by LFQ in at least one sample per buffer were considered. Independently of the number of unique peptides, a total of 574, 814, and 858 proteins were quantified with RIPA, Urea/thiourea, and HEPES LBs, respectively (Figure 7A). Regarding the proteins quantified with at least two unique peptides, 542, 774, and 814 proteins were found, showing that HEPES buffer allowed the quantification of a higher number of proteins (Figure 7D). However, when normalizing these results to the total number of identified proteins, a similar proportion of proteins identified with ≥2 unique peptides was observed between the three buffers (RIPA, 94% (Figure 7B); Urea/thiourea, 95% (Figure 7C); and HEPES, 95% (Figure 7D). Hence, for studies involving quantitative analysis, there is no clear preference between the three buffers, as all yielded a similar percentage of confident protein identifications. This suggests that the selection of a buffer should rather account for the compatibility with downstream applications.

**FIGURE 7:**
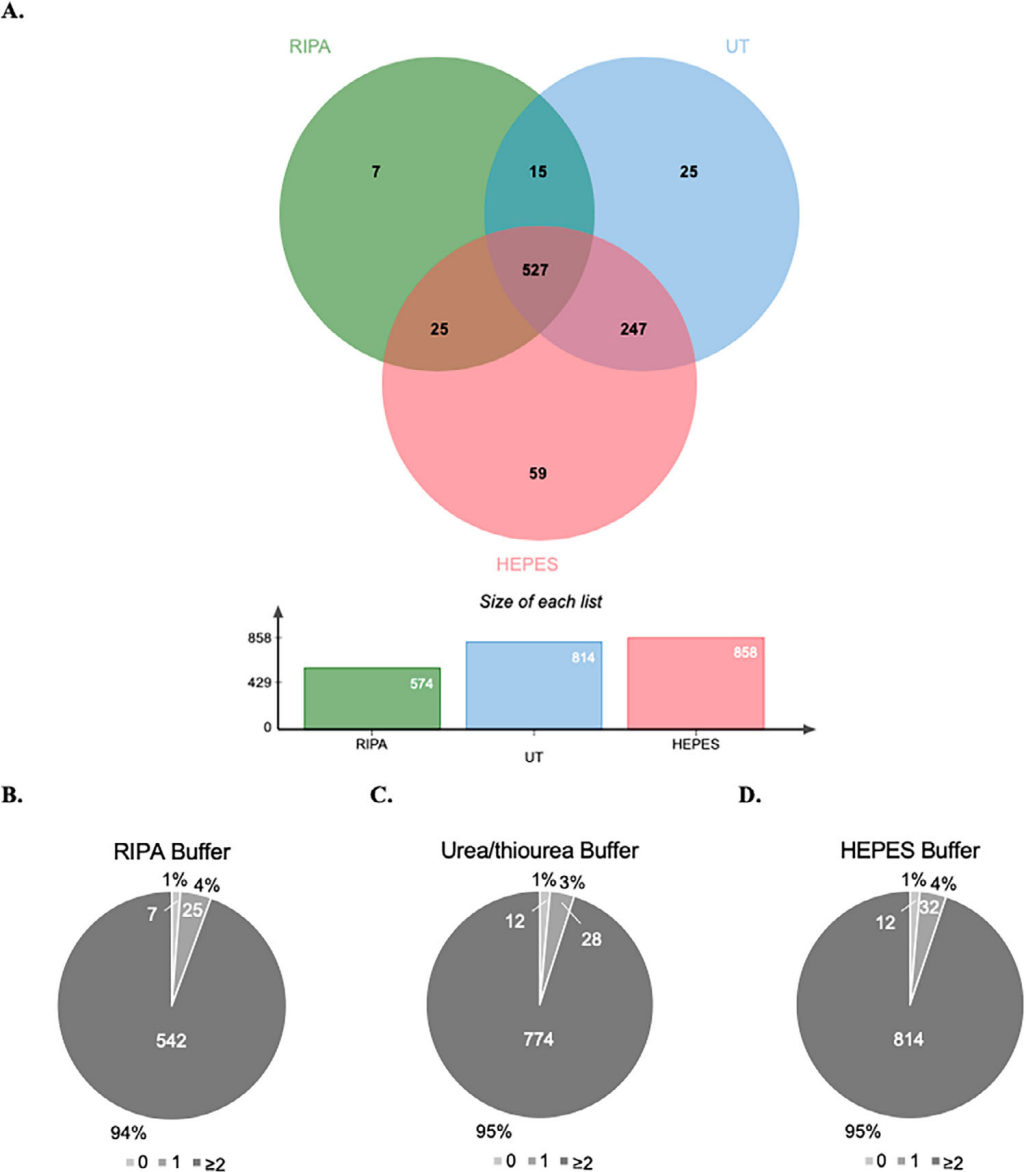
Protein quantification performance by LC‐MS/MS. (A) Venn diagram presenting the number of quantified proteins, with at least two unique peptides, for each buffer (RIPA, Urea/thiourea (UT), and HEPES); Pie charts illustration with the distribution of proteins quantified with 0, 1, or ≥2 unique peptides, using RIPA (B), Urea/thiourea (C), and HEPES (D) buffers.

Sequence coverage (SC) is another parameter that helps evaluate the confidence in protein identification, as it reflects the percentage of a protein's amino acid sequence that is identified through MS/MS. For this study, the analysis of SC was performed for proteins with at least two peptides identified (Figure 8A). Notable differences in the performance of the buffers were observed. RIPA buffer was associated with a lower proportion of identified proteins with a SC below 20%, explained by a higher proportion of proteins with a SC greater than 30%, compared to the other buffers. These results suggest that RIPA is more effective in solubilizing tissue‐extracted proteins and digestion‐derived peptides, resulting in a greater distribution across the primary sequence, which can be advantageous when researchers seek more in‐depth knowledge of the protein domains, modifications, and functional aspects of the identified proteins.

**FIGURE 8:**
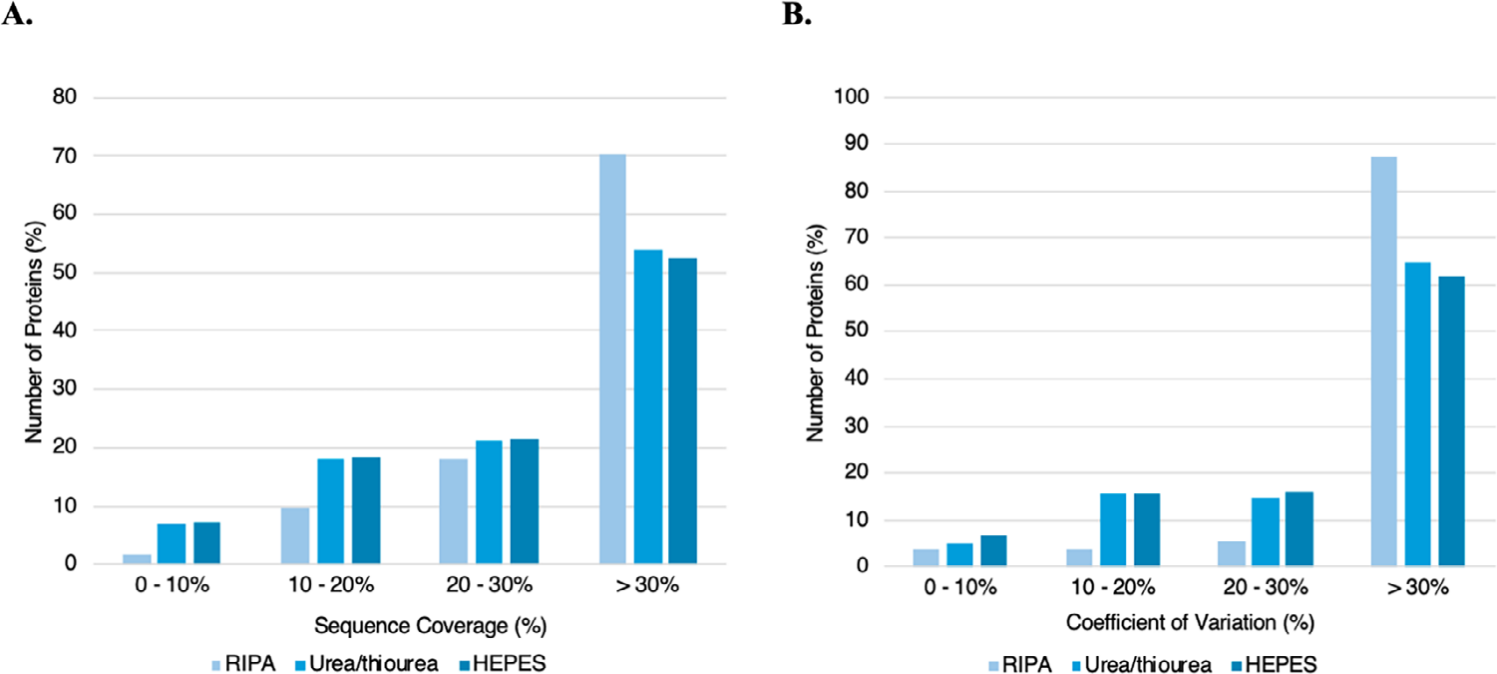
(A) Distribution of the number of proteins identified according to the sequence coverage using RIPA, Urea/thiourea, and HEPES buffers. Sequence coverage is defined as the percentage of the amino acid residues sequentially identified of the total number of amino acid residues in the protein sequence. (B) Distribution of the number of proteins identified according to the coefficient of variation (%) using RIPA, Urea/thiourea, and HEPES lysis buffers. The coefficient of variation is a statistical measure of relative variability expressed as the ratio of SD to the mean.

In order to identify the LBs that could minimize technical variance, we determined the proteins’ coefficient of variation (CV) regarding LFQ. Since we used the same three biological replicates in three LB extractions, a similar degree of biological variance is expected, and any difference between the LBs should be mostly explained by the technical variance. A lower CV indicates lower technical variability and a higher potential for reproducibility. As shown in Figure 8B, Urea/thiourea and HEPES buffers showed a consistently higher number of proteins with a CV < 30% than the RIPA buffer, and a lower number of proteins identified with a CV > 30%. Hence, these results are suggestive that protein extraction with Urea/thiourea or HEPES leads to more reproducible proteomic quantifications.

Given the substantial differences in protein identifications between all extraction buffers, we investigated whether these variations reflect the identification of proteins participating in different processes and pathways. To further characterize proteins identified in different extractions, a functional enrichment analysis was performed.

GO analysis was performed to evaluate the biological function associated with the proteins extracted from aneurysmatic tissue using the previously mentioned LBs (RIPA, Urea/thiourea, and HEPES). The top 15 BPs for each condition are ranked in Figure 9. Common to the three buffers were eight BPs, including blood coagulation, wound healing, immune system process, or regulation of peptidase activity. Regarding the processes exclusively identified in each condition, DNA replication‐dependent chromatin assembly and organization, cellular detoxification, and biological process involved in interspecies interaction between organisms were highlighted for RIPA, Urea/thiourea, and HEPES, respectively. In the CC subontology, the top 15 GO terms for each buffer (Figure S3) revealed a similar enrichment of the terms among the buffers, with a highlight of the collagen‐containing ECM as a common CC to RIPA and Urea/thiourea buffer, which can be relevant in the context of ECM remodeling in AAA. Regarding the MF category (Figure S4), a similar enrichment profile was observed, with antioxidant activity being highlighted as a common MF to RIPA and Urea/thiourea buffers, which is also relevant in the context of AAA pathology. In summary, slight differences in GO terms for BP, CC, and MF were observed for the three LBs. Figures S3 and S4 are availbale in the Supporting File S2.

**FIGURE 9:**
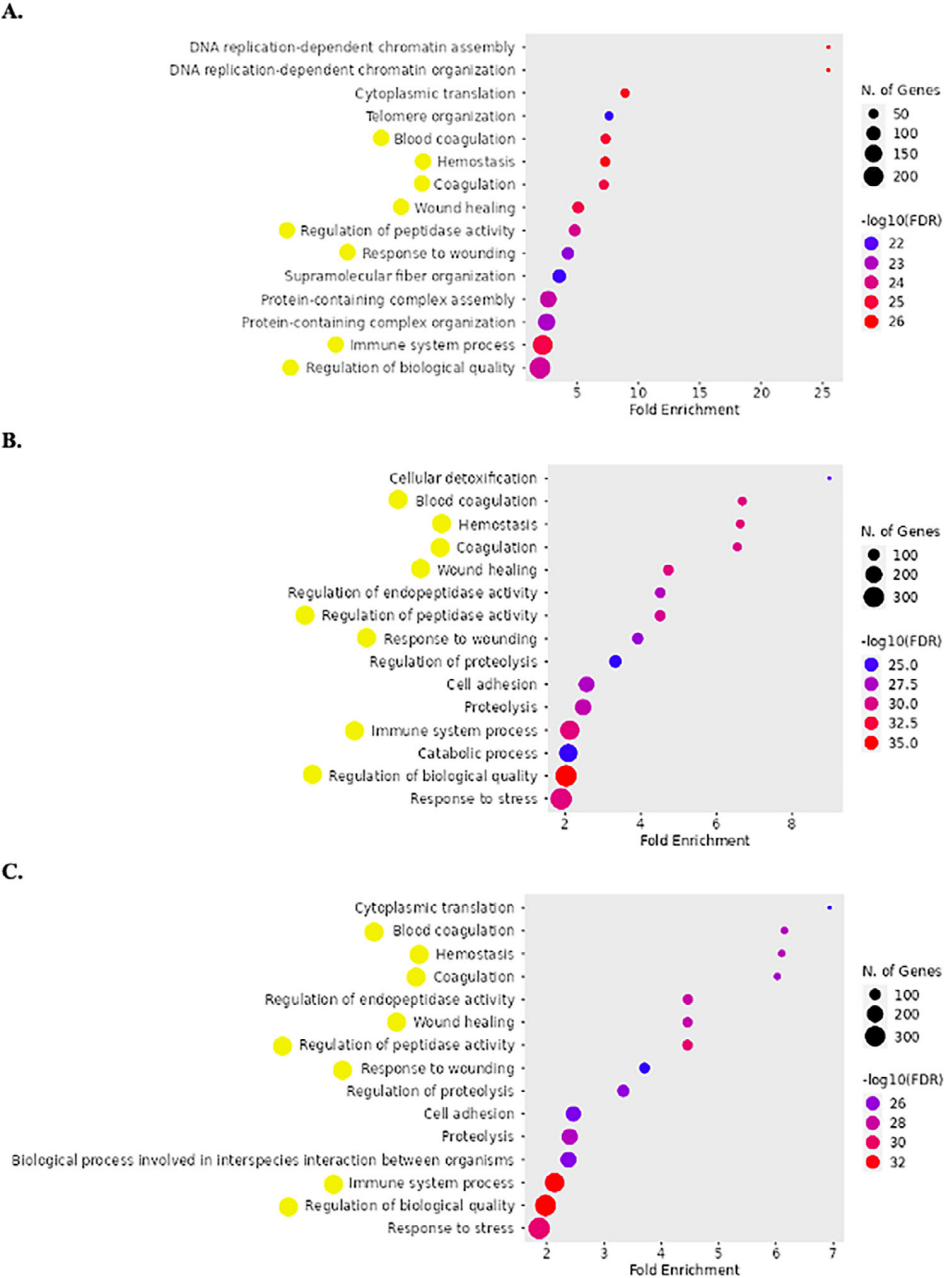
GO enrichment—Biological Processes. Top 15 BPs of the proteins identified using RIPA (A), Urea/thiourea (B), and HEPES (C) LBs. The size of the dots is proportional to the number of mapped proteins/genes, and the dots’ color represents the degree of significance. Common processes to the three analyses are highlighted by the yellow circles. Plots were created with the ShinyGO online tool.

Reactome analysis was also performed to further assess the pathways that can be studied when extracting the proteins from aneurysmatic tissue with different LBs (Figure 10). Many pathways were consistently highlighted with the different buffers, such as neutrophil degranulation (R‐HSA‐6798695), cellular responses to stress (R‐HSA‐2262752), and to stimuli (R‐HSA‐8953897), metabolism of proteins (R‐HSA‐392499), and pathways related to the immune system (R‐HSA‐168256). These findings are consistent with several pathways reported in a recent literature review on AAA pathophysiology [8]. In the context of AAA pathophysiology, the aortic wall weakening is driven by ECM remodeling and degradation, leading to stimulation of cell death and inflammation, primarily mediated by Matrix metallopeptidase 12 (MMP‐12) [21]. Beyond the top 15 pathways identified in the Reactome enrichment analysis (data not shown), the ECM organization (R‐HSA‐1474244) pathway ranked within the top 25 across all three buffers analyzed, with 58, 73, and 73 assigned proteins for RIPA, Urea/thiourea, and HEPES buffers. In all cases, MMP‐12 was present in the set list of the identified proteins. Complementary analysis further confirmed ECM degradation, though it was only among the top 100 pathways in the Reactome analysis for the Urea/thiourea buffer. Platelets also play a pivotal role in AAA progression, as their activation and degranulation exacerbate vascular inflammation, promote a thrombogenic environment, and contribute to ECM remodeling [22]. The platelet activation, signaling, and aggregation pathway (R‐HSA‐76002) ranked within the top 31 for all buffer analysis, with 54 proteins identified for RIPA and 67 proteins for Urea/thiourea and HEPES buffers. Additionally, the platelet degranulation pathway (R‐HSA‐114608) was among the top 20 pathways for RIPA (43 proteins) and Urea/thiourea (54 proteins). Since apoptosis also accelerates ECM and aortic tissue degradation [23], the apoptosis pathway (R‐HSA‐109581) was searched and ranked within the top 100 for HEPES (33 proteins) and Urea/thiourea (32 proteins). To summarize, the enrichment analysis highlighted ECM remodeling as a key pathway across all buffer conditions, while platelet activation and degranulation were strongly represented, reinforcing their contribution to vascular inflammation and aneurysm progression.

**FIGURE 10:**
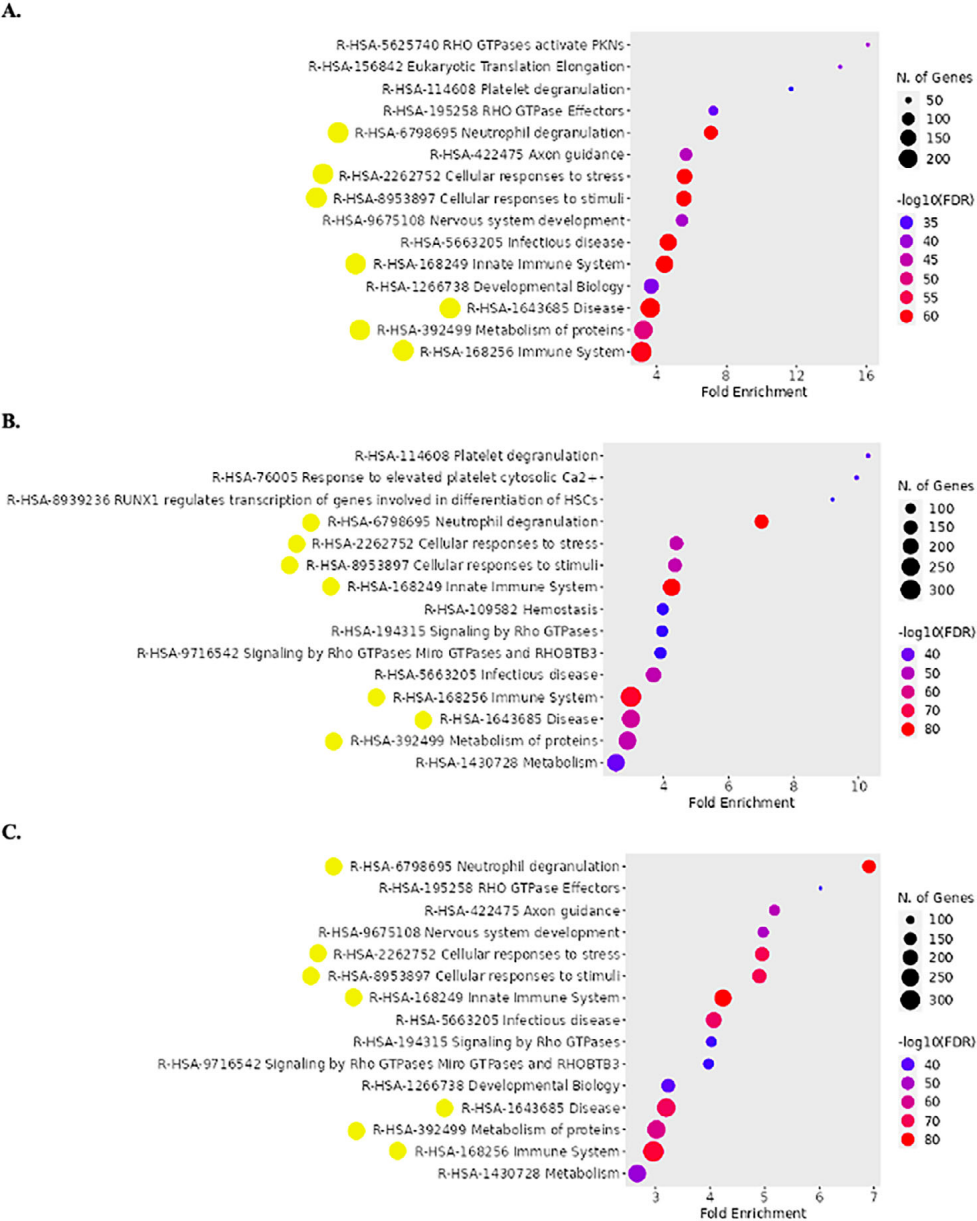
Reactome pathways enrichment. Top 15 curated Reactome pathways presented for each condition of protein extraction from aneurysmatic tissue using RIPA (A), Urea/thiourea (B), and HEPES (C) as LBs. Common pathways to the three analyses are highlighted by the yellow circles nearby. Dot plots were created with the ShinyGO online tool. R‐HSA, Reactome‐*Homo sapiens*.

Of note, in the present workflow, we focused on the analysis of the soluble protein fraction. However, after centrifugation, a pellet is also obtained. This fraction is likely enriched in insoluble proteins, including membrane‐bound components and ECM proteins, which play an undeniable role in AAA pathobiology, namely, during vascular remodeling and ECM degradation during aneurysm progression. Only a few protocols have been established for the extraction of proteins from insoluble material from human aortic tissue, aiming to broaden the proteomic landscape of the aneurysm [11, 24]. It is likely that our standardized protocol might also improve the proteomic characterization of this fraction. However, we have not focused on insoluble proteins, and therefore this remains a limitation of the present work. Future refinements that integrate complementary extraction strategies will enhance the depth, reproducibility, and biological interpretability of AAA proteomic profiling.

A second important limitation of our protocol is that we limited to bulk AAA tissue analysis. Spatial proteomics approaches such as those targeting the intraluminal thrombi or focused on the extracellular matrix may give more focused and complementary mechanistic insights into AAA pathophysiology [11, 25]. However, because they require additional fractionation and sample treatment steps, which can imperil reproducibility, we decided to focus only bulk thrombus‐resected aortic wall tissue, requiring minimal processing and thereby providing a simpler proteomic workflow to maximize reproducibility, which can be especially relevant for large cohort biomarker studies.

In conclusion, we have developed an SOP for optimal and more reproducible homogenization of AAA tissue and protein extraction. Protein extraction was best when using 1.4 mm zirconium dioxide beads, in a proportion of 30 × the tissue mass, using 20 µL of lysis buffer per mg of tissue, and with no more than two homogenization cycles. The lysis buffers presented comparable performance, and the choice between them should account for the main goal of the downstream analysis. HEPES and Urea/thiourea buffers demonstrated superior performance in terms of quantification reproducibility, as reflected in their consistently low CV scores. Conversely, the RIPA buffer exhibited better performance in ensuring reproducibility during protein extraction. Regarding the potential for discovery, no significant differences were definitively observed between the buffers, as the optimal choice ultimately depends on the specific application and focus of the study being undertaken. Overall, this protocol was successfully applied to the characterization of the tissue proteome in patients presenting AAA, and its robustness and reproducibility in extracting and identifying proteins from this highly heterogeneous tissue were demonstrated. Given the importance of optimized conditions of protein extraction from AAA tissue for downstream proteomics applications and biomarker discovery, this SOP will be made available as a supplement to this publication to provide researchers with a detailed guide for implementing the protocol in similar proteomic experimental contexts.

## Author Contributions

**Telmo Baltazar:** writing – original draft, investigation, methodology, formal analysis. **Fábio Trindade:** writing – review and editing, methodology, supervision. **Rita Nogueira‐Ferreira:** writing – review and editing, methodology; **Rui Vitorino:** writing – review and editing. **Rita Ferreira:** writing – review and editing. **Pedro Domingues:** writing – review and editing. **Adelino Leite‐Moreira:** writing – review and editing. **Marina Dias‐Neto:** writing – review and editing, conceptualization, funding acquisition, supervision. **VASCUL‐AID Consortium:** writing – review and editing, project administration.

## Ethics Statement

This study was conducted according to the guidelines of the Declaration of Helsinki, and approved by the Ethics Committee of Centro Hospitalar Universitário São João (reference CE‐159‐2014).

## Consent

Informed consent was obtained from all the participants involved in this study.

## Conflicts of Interest

The authors declare no conflicts of interest.

## Supporting information




**Supporting File 1:** prca70030‐sup‐0001‐SF1.docx.

**Supporting File 2:** prca70030‐sup‐0002‐SF2.docx.

## Data Availability

The mass spectrometry proteomics data have been deposited to the ProteomeXchange Consortium via the PRIDE [26] partner repository with the dataset identifier PXD060417.
