# Supplementary material for: A Standard Operating Procedure for Protein Extraction From Abdominal Aortic Aneurysm Tissue: Enhancing Proteomics Applications
Source: Proteomics Clin Appl. 2025 Nov 14;20(1):e70030. doi: 10.1002/prca.70030 (PMC12743587; doi:10.1002/prca.70030)
Supplement: Supplementary file 1 — Supporting File 1: prca70030‐sup‐0001‐SF1.docx. [file PRCA-20-e70030-s002.docx]

Index

[Introduction 2](#_Toc66959519)

[Aims & scope 2](#_Toc66959520)

[Abbreviations 2](#_Toc66959521)

[Consumables 2](#_Toc66959522)

[Reagents 3](#_Toc66959523)

[Materials & equipment 3](#_Toc66959524)

[Protocol 4](#_Toc66959525)

# Introduction

This protocol is the standard procedure used in the Department of Surgery and Physiology of the Faculty of Medicine of the University of Porto (RISE-Health) for the homogenization of abdominal aortic aneurysm (AAA) tissue and subsequent protein extraction. A state-of-the-art bead-beating system with zirconium oxide beads is used to fragment the tissue, in conjunction with a lysis buffer, enriched with phosphatase and protease inhibitors, which promotes cell lysis and protein solubilization. A centrifugation step ensures the purification of a protein-rich supernatant, and the precipitation of cell debris. Protein concentration is estimated using a detergent-compatible protein assay kit.

# Aims & scope

The purpose of this SOP is to standardize the procedures for protein extraction from AAA tissue aiming at increasing the reproducibility of downstream analysis, particularly, proteomic analysis, with especial emphasis on biomarker discovery, and protein analysis by standard immunoassays, such as western blot or similar techniques.

# Abbreviations

| Acronym/abbreviation | Definition |
| --- | --- |
| AAA | Abdominal aortic aneurysm |
| CHAPS | 3-((3-cholamidopropyl) dimethylammonio)-1-propanesulfonate |
| DC | Detergent compatible |
| EDTA | Ethylenediaminetetraacetic acid |
| HEPES | 4-(2-hydroxyethyl)-1-piperazineethanesulfonic acid |
| LB | Lysis buffer |
| NP-40 | Nonidet P-40 |
| PhIC | Phosphatase inhibitor cocktail |
| PIC | Protease inhibitor cocktail |
| RT | Room temperature |
| SDS | Sodium dodecyl sulfate |
| UW | Ultrapure water |

# Consumables

| Name | Company | Reference | Storage conditions | Localization |
| --- | --- | --- | --- | --- |
| Precellys ® Zirconium oxide beads (1.4 mm) | Bertin Technologies | 23405-300-RD001 | RT | Molecular Biology lab |
| Screw-cap O-ring 2 mL microtubes | Sarstedt | 72.694.006 | RT | Molecular Biology lab |

# Reagents

| Name | Company | Reference | Storage conditions | Localization |
| --- | --- | --- | --- | --- |
| UW (Simplicity® Water Purification System) | Merck - Millipore | - | RT | Molecular Biology Lab |
| Ice | - | - | - | Washing room |
| Halt™ PIC, EDTA-Free 100x | Thermo Fisher  Scientific | 78439 | 4 °C | Cold room |
| PhosSTOP^TM^ (PhIC) | Roche | 4906837001 | 4 °C | Cold room |
| EDTA disodium salt dihydrate (≥99%) | Merck - Millipore | 1084540100 | RT | Mass Spectrometry Lab |
| Urea (99.5%) | acros organics | 327380010 | RT | Mass spectrometry cabinet |
| Thiourea (≥99.0%) | Sigma-Aldrich | T7875-500G | RT | Mass Spectrometry Lab |
| Tris Base | Fisher Bioreagents | BP152-1 | RT | Chemistry Lab |
| CHAPS | Calbiochem, Sigma-Aldrich | 3050-10GM | RT | Mass Spectrometry Lab |
| HEPES | VWR Chemicals | 441487M | RT | Chemistry Lab |
| Sarcosyl (N-lauroylsarcosine sodium salt) | Sigma-Aldrich | 61745-50G | RT | Mass Spectrometry Lab |
| Sodium dodecyl sulfate (SDS) | Fisher Bioreagents | BP8200-500 | RT | Chemistry Lab |
| DC Protein Assay kit | Bio-Rad | 5000116 or 5000120 | RT | Mass Spectrometry Lab |

# Materials and Equipment

| Name | Notes |
| --- | --- |
| Precision scale | Maximal mass difference ± 0.01 g |
| Analytical scale | Maximal mass difference ± 0.1 mg |
| Vortex |  |
| Liquid nitrogen container |  |
| Mortar and pestle | ~12cm diameter mortar |
| Spatulas and tweezers |  |
| Bead-beating system, Bertin Instruments | The protocol has been optimized using the Precellys Tissue Homogenizer (Precellys^®^ Evolution Touch, 23405-300-RD001, Bertin Technologies, Montigny-le-Bretonneux, France) |
| Chronometer |  |
| Benchtop centrifuge | Refrigerable, compatible with 2 mL and 1.5mL tubes, and reaching 12,000 rpm |
| Minicentrifuge | To spin down lysates |

# Protocol

1. Label one O-ring 2 mL tube, and at least three 1.5 mL tubes (one for centrifugation, one for quantification and another for storage) for each sample.

*Should you wish to aliquot the sample, label more tubes.*

1. In another set of 2 mL tubes weigh the zirconium oxide beads. The amount should be 30× the amount of tissue to homogenize. Fill as many tubes as the number of samples to process. Fill an extra tube with beads, just in case some sample exceeds greatly the initially planned amount.

*This protocol has been optimized for 15 mg of starting material, requiring 0.45g of beads.*

*To prevent precipitation, the urea/thiourea buffer should not be buried deep in the ice.*

1. Prepare the lysis buffer (LB):

| Lysis Buffer | Chemical Composition |
| --- | --- |
| RIPA (commercial solution) | 25 mM Tris-HCl pH 7.6, 150 mM NaCl, 1% NP-40, 1% sodium deoxycholate, 0.1% SDS, 1 mM EDTA |
| Urea/thiourea | 8 M urea, 2 M thiourea, 45 mM Tris-HCl pH 7.6, 4% CHAPS,1 mM EDTA |
| HEPES | 25 mM HEPES, 1% sarcosyl, 1 mM EDTA |

All buffers were enriched with protease (PIC) and phosphatase (PhIC) inhibitors.

*The following sub-steps are designed for a total of* ***10 mL lysis buffer****, which is enough to process ~30 AAA samples weighing around ~15 mg.*

*Preparation of the buffers:*

- *Urea/Thiourea Buffer:*
  - *Weigh 12.01 g of urea in a beaker and tare the balance. Add 3.81 g of thiourea and tare again. Incorporate 136 mg of Tris Base. Due to the high molarity of the solution, add approximately 7 mL of ultrapure water (UW) and allow it to dissolve under agitation. Measure the pH and adjust it to 8.0. Bring the final volume to 25 mL. From this solution, take 10 mL and add 400 mg of CHAPS. Finally, take 9.880 mL of the solution to complete the lysis buffer and add the EDTA, PhIC and PIC as described in steps 3.1., 3.2., and 3.5., respectively.*
- *HEPES Buffer:*
  - *Weigh 298 mg of HEPES and dissolve it in ultrapure water (UW). Adjust the final volume to 50 mL. Take 8 mL of the solution and measure the pH. Adjust the pH to 7.4, then add 100 mg of sarcosyl and bring the volume to 10 mL. Finally, take 9.880 mL of the solution to complete the lysis buffer and add the EDTA, PhIC and PIC as described in steps 3.1., 3.2., and 3.5, respectively.*
  1. In an empty 15mL Falcon tube, add 20 µL of a 1 mM EDTA stock solution.
  2. Add a PhIC tablet.

*Each tablet should be dissolved in 10 mL of solution. It is recommended to prepare the lysis buffer in multiples of 10 mL.*

- 1. Add 9.880 mL of cold lysis buffer to the Falcon tube.

*Important note: in the case of using the Urea/Thiourea buffer, exposure to low temperatures is not recommended due to the risk of urea and thiourea precipitation.*

- 1. Vortex until all reagents are solubilized.

*For 10 mL, this step takes less than one minute.*

- 1. Add 100 µL of cold PIC.
  2. Close the lid, homogenize by gently inverting ten times.
  3. Keep the LB on ice (if applicable).

1. Fill a styrofoam box with ice. Keep the lysis buffer, PIC and PhIC on ice.
2. Start refrigerating the centrifuge to 4°C.
3. Fill a canister with liquid nitrogen and add up to 6 samples to it.
4. Disinfect the lab bench around the scale with ethanol 70%.
5. Disinfect the mortar, pestle, and tweezers with ethanol 70%.
6. Take one sample cryotube, at a time, from the nitrogen canister and place it immediately on ice.
7. Tare the respective O-ring tube containing the beads, lid off, in the analytical scale.
8. Add some nitrogen to the mortar and cool the pestle.
9. Quickly transfer the sample to the mortar and break it down.
10. After obtaining a piece of ~15 mg, transfer the remaining sample back into the cryotube, and place it again on ice.

*15 mg of AAA tissue corresponds roughly to a 6-7mm x 6-7mm piece.*

1. Add more nitrogen to break into smaller pieces of 2-3 mm and transfer them to the O-ring tube. Register the weight.

*Do not over-fragment the sample, as this leads to major sample loss and to quick defrosting. However, note that the efficiency of protein extraction increases if smaller sample portions are obtained.*

1. Add the LB (20 µL/mg tissue) and using the pipette tip immerse any dry tissue fragments, attached to the tube wall.

*The LB is easily calculated as 20 times the amount of tissue, e.g., for 15 mg, 300 µL of buffer is required.*

1. Add the corresponding beads. By every 2 mg of additional tissue, add one extra bead (~ 0.04g). Close the lid and quickly place the tube on ice.
2. Tare the next O-ring tube.
3. Store the remaining unprocessed sample back in the canister. Take the next sample and keep it on ice.
4. Repeat steps 9-18 and proceed until all samples have been homogenized.

*Since some AAA samples are harder than others to break down, due to the heterogeneity of the tissue, some samples take longer to process. For that reason, is preferable to keep them in the freezer, and take batches of samples to the nitrogen canister and take one at time to prevent unwanted defrosting.*

1. Homogenize the samples in the Precellys Homogenizer instrument, at maximum speed (5,000 rpm), for 30 seconds.

*Efficient extractions are also possible using the Bead Mill Cryo (Omni International), at maximum speed (6.5 m/s), for 30 seconds.*

1. Cool the samples down in ice for 5 minutes.
2. Repeat step 20, and put the samples back on ice.

*Attention: this step is dependent on the instrument and sample rigidity. If needed, this step can be repeated twice, should one find undisrupted tissue. In any case, do not homogenize for more than 3 times.*

1. Spin down the samples for one minute, using the minicentrifuge.

*If there is still too much foam in some samples, repeat this step in 30 seconds increments.*

1. Transfer the homogenate to an empty 1.5mL tube.
2. Centrifuge the samples at 13,680 × *g* for 15 minutes at 4 °C.
3. Transfer the supernatant to an empty 1.5mL tube. Discard the pellet or store it at -80 ºC if you would like to perform other analyses.

*Pellet analysis may provide insights into extracellular matrix or membrane-associated proteins.*

1. Save at least 5 µL for protein quantification.
2. Save the remaining LB at 4°C for the preparation of the protein standards for quantification in the same or next day.
3. Estimate the protein concentration using the DC assay, following the manufacturer’s instruction.

*The expected protein concentrations range between 1-5 µg/µL. Dilute the samples 5 and 10 times. For quantification, it is recommended to collect 3 µL of the protein lysate and dilute with 27 µL of ultrapure water.*

**Additional Protocol – Protein Precipitation for Protein Quantification**

When using the urea/thiourea buffer, there is a risk of protein estimation with the DC assay. Therefore, protein should be precipitated before quantification.

1. Determine the volume of sample that will be used for protein precipitation.

*We expect a protein concentration of ~3 µg/µL. 20 µL is recommended to precipitate ~60 µg of protein.*

1. To each eppendorf, add 9 parts (in volume) of cold acetone to 1 part of tissue lysate.

*In this case, add 180 µL of cold acetone on top of 20 µL of lysate.*

1. Precipitate the protein at -20 ºC, for at least, 2h to overnight.
2. Centrifuge at 14000 *xg* for 30 minutes at 4 ºC.
3. Discard the supernatant and wash the pellet with 90% ice-cold ethanol, using the same volume as acetone.

*Washing with ethanol facilitates the removal of urea/thiourea, which can lead to an overestimation of protein concentration using the DC assay.*

1. Remove the supernatant and resuspend the pellet in 50 µL SDS 0.1%.
2. Heat the samples at 40 ºC for 15 minutes in the heating block, with slow agitation.
   1. Vortex and spin down.
3. Collect 5-30 µL for quantification and proceed with the DC assay.
