# Supplementary material for: A Standard Operating Procedure for Protein Extraction From Abdominal Aortic Aneurysm Tissue: Enhancing Proteomics Applications
Source: Proteomics Clin Appl. 2025 Nov 14;20(1):e70030. doi: 10.1002/prca.70030 (PMC12743587; doi:10.1002/prca.70030)
Supplement: Supplementary file 2 — Supporting File 2: prca70030‐sup‐0002‐SF2.docx. [file PRCA-20-e70030-s001.docx]

**Supplementary Material**

**
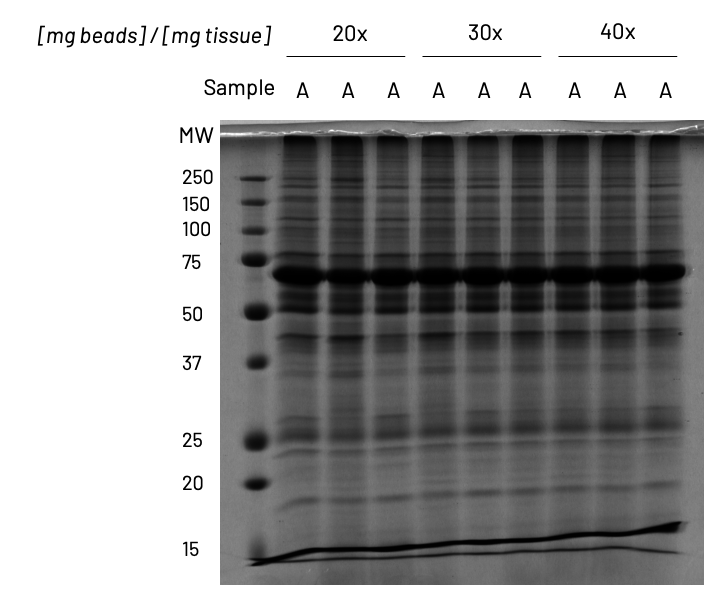
**

**Supplementary Figure 1.** SDS-PAGE profile of the AAA tissue lysates obtained after protein extraction with different ratios (20, 30 or 40) of beads mass-to-tissue mass (mg/mg). Twenty micrograms (μg) of protein were loaded in the gel. Samples designated with “A” stands for a unique biological AAA sample. MW: Molecular weight.

**
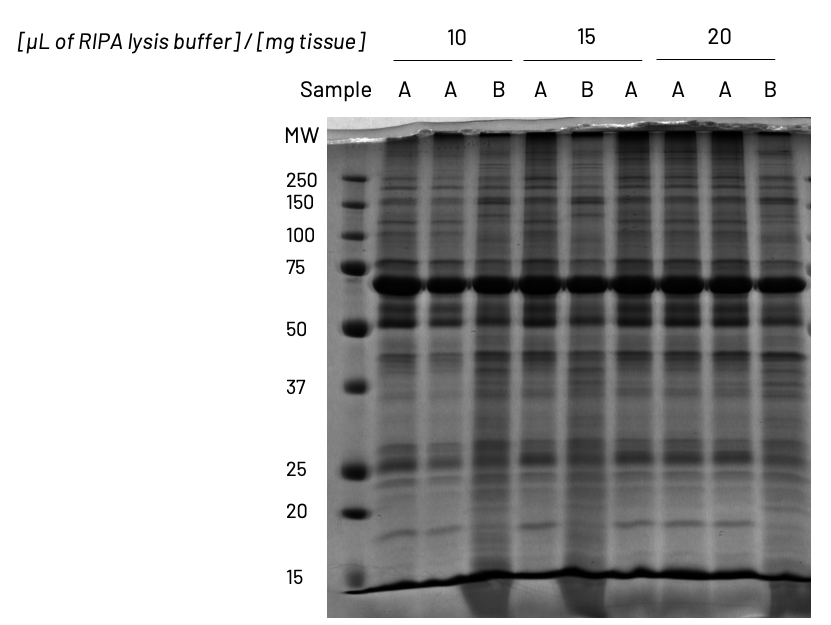
**

**Supplementary Figure 2.** SDS-PAGE profile of the AAA tissue lysates obtained after protein extraction with different ratios (10, 15 or 20) of volume of lysis buffer-to-tissue mass (μL/mg). Twenty micrograms (μg) of protein were loaded in the gel. A, and B refer to different biological AAA samples. MW: Molecular weight.


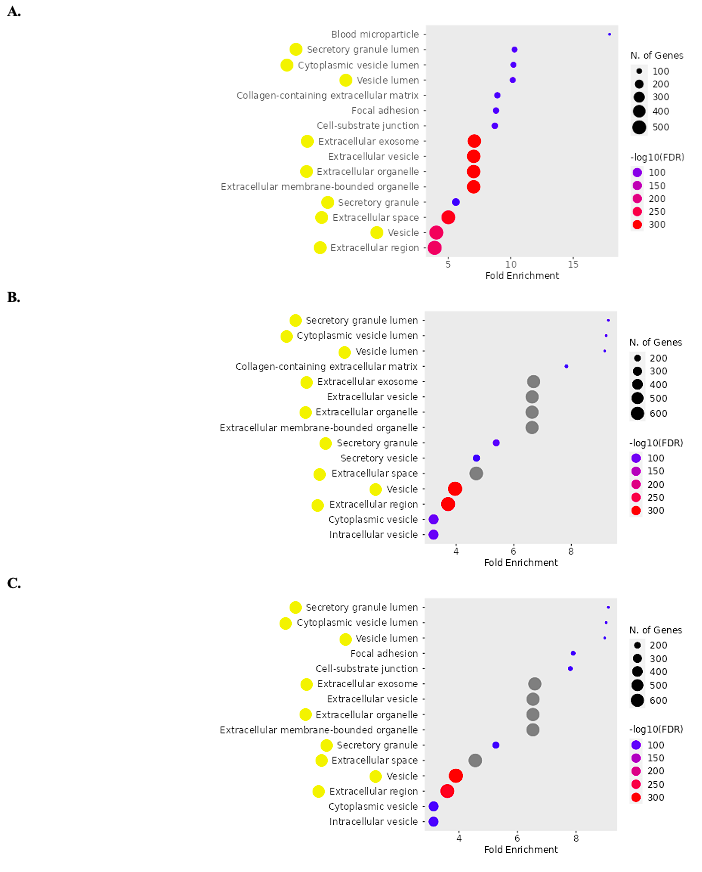


**Supplementary Figure 3.** GO enrichment - Cellular Components. Top 15 cellular components (CC) presented for each condition of protein extraction from aneurysmatic tissue using RIPA **(A.)**, Urea/thiourea **(B.)**, and HEPES **(C.)** as LBs. Common cellular components to the three analyses are highlighted by the yellow circles nearby. Dot plots were created with ShinyGO online tool. R-HSA, Reactome-*Homo sapiens*.

**
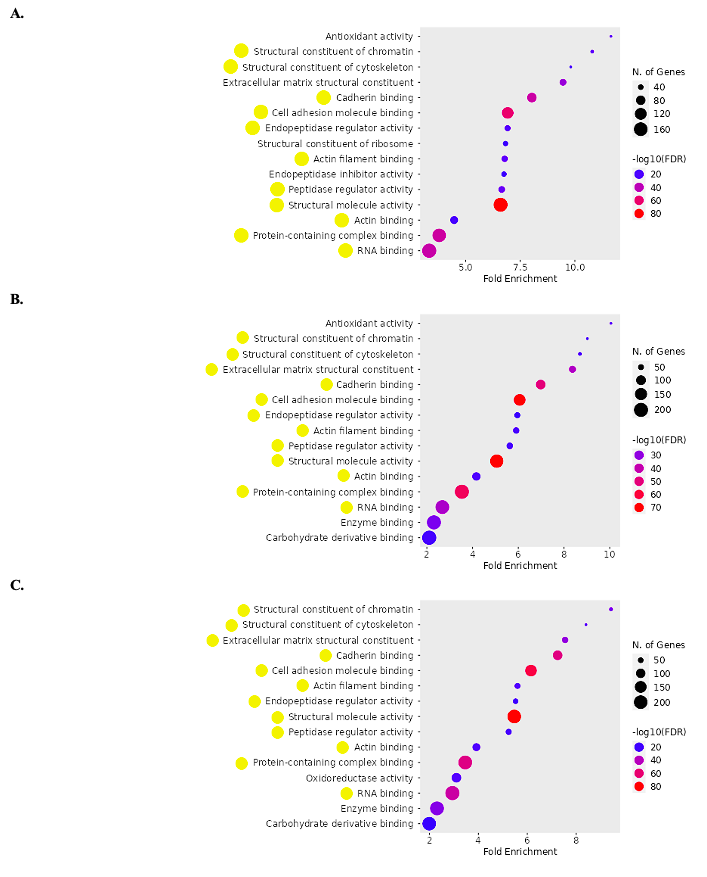
**

**Supplementary Figure 4.** GO enrichment - Molecular Functions. Top 15 molecular functions (MF) presented for each condition of protein extraction from aneurysmatic tissue using RIPA **(A.)**, Urea/thiourea **(B.)**, and HEPES **(C.)** as LBs. Common molecular functions to the three analyses are highlighted by the yellow circles nearby. Dot plots were created with ShinyGO online tool. R-HSA, Reactome-*Homo sapiens*.
